# Supplementary material for: Stratified microbial communities in Australia’s only anchialine cave are taxonomically novel and drive chemotrophic energy production via coupled nitrogen-sulphur cycling
Source: Microbiome. 2023 Aug 26;11:190. doi: 10.1186/s40168-023-01633-8 (PMC10463829; doi:10.1186/s40168-023-01633-8)
Supplement: Supplementary file 2 — Additional file 1: Figure S1. Correlation between dissolved organic carbon and nitrification. (a) Correlation between the relative abundance (TPM) of the marker gene for ammonia oxidation (amoA; first step of nitrification) and dissolved organic carbon (DOC) concentration. (b) Correlation between the relative abundance of the marker gene for nitrite oxidation (nxrA; final step of nitrification) and DOC concentration. Shaded regions represent the 95% confidence interval of the fitted linear model. A full list of r2 and p-values for all evaluated nitrogen and sulphur cycling gene correlations is presented as Supplementary Table 5. Figure S2. Beta-diversity of MAG phyla in the Bundera sinkhole. Non-metric multidimensional scaling (NMDS) based on Bray-Curtis distances of the relative abundance for MAG phyla. NMDS points that represent replicate samples lie on top of each other, as do those representing all samples from 17, 18, 22, and 28 m depths. The groupings (circles, triangles, and squares) represent samples with similar levels of dissolved oxygen (DO) and salinity (Supplementary Table 2). The grouping of samples from 17, 18, 22, and 28m depths (squares) is supported by PERMANOVA (p=0.046; Supplementary Table 7). [file 40168_2023_1633_MOESM1_ESM.docx]

**Supplementary Figures**

**Fig. S1. Correlation between dissolved organic carbon and nitrification.** (**a**) Correlation between the relative abundance (TPM) of the marker gene for ammonia oxidation (*amoA*; first step of nitrification) and dissolved organic carbon (DOC) concentration. (**b**) Correlation between the relative abundance of the marker gene for nitrite oxidation (*nxrA*; final step of nitrification) and DOC concentration. Shaded regions represent the 95% confidence interval of the fitted linear model. A full list of r^2^ and p-values for all evaluated nitrogen and sulphur cycling gene correlations is presented as Supplementary Table 5.

**Fig. S2. Beta-diversity of MAG phyla in the Bundera sinkhole**. Non-metric multidimensional scaling (NMDS) based on Bray-Curtis distances of the relative abundance for MAG phyla. NMDS points that represent replicate samples lie on top of each other, as do those representing all samples from 17, 18, 22, and 28 m depths. The groupings (circles, triangles, and squares) represent samples with similar levels of dissolved oxygen (DO) and salinity (Supplementary Table 2). The grouping of samples from 17, 18, 22, and 28m depths (squares) is supported by PERMANOVA (p=0.046; Supplementary Table 7).
